# Exposure to Polypropylene Microplastics via Oral Ingestion Induces Colonic Apoptosis and Intestinal Barrier Damage through Oxidative Stress and Inflammation in Mice

**DOI:** 10.3390/toxics11020127

**Published:** 2023-01-28

**Authors:** Rui Jia, Jie Han, Xiaohua Liu, Kang Li, Wenqing Lai, Liping Bian, Jun Yan, Zhuge Xi

**Affiliations:** 1 College of Marine Ecology and Environment, Shanghai Ocean University, Shanghai 201306, China; 2 Tianjin Institute of Environmental and Operational Medicine, Tianjin 300050, China

**Keywords:** polypropylene microplastics (PP-MPs), intestinal barrier, oxidative stress, inflammatory reaction, apoptosis

## Abstract

Extensive environmental pollution by microplastics has increased the risk of human exposure to plastics. However, the biosafety of polypropylene microplastics (PP-MPs), especially of PP particles < 10 μm, in mammals has not been studied. Thus, here, we explored the mechanism of action and effect of exposure to small and large PP-MPs, via oral ingestion, on the mouse intestinal tract. Male C57BL/6 mice were administered PP suspensions (8 and 70 μm; 0.1, 1.0, and 10 mg/mL) for 28 days. PP-MP treatment resulted in inflammatory pathological damage, ultrastructural changes in intestinal epithelial cells, imbalance of the redox system, and inflammatory reactions in the colon. Additionally, we observed damage to the tight junctions of the colon and decreased intestinal mucus secretion and ion transporter expression. Further, the apoptotic rate of colonic cells significantly increased after PP-MP treatment. The expression of pro-inflammatory and pro-apoptosis proteins significantly increased in colon tissue, while the expression of anti-inflammatory and anti-apoptosis proteins significantly decreased. In summary, this study demonstrates that PP-MPs induce colonic apoptosis and intestinal barrier damage through oxidative stress and activation of the TLR4/NF-κB inflammatory signal pathway in mice, which provides new insights into the toxicity of MPs in mammals.

## 1. Introduction

The output of plastic and related products is continuously increasing because of its widespread use and low cost. It is estimated that the total output of plastic waste will reach 12 billion tons in 2050 [1]. Plastic use in the past few decades has caused environmental issues through the lack of sound recycling and treatment measures. Widespread plastic pollution is considered to be a global threat to human and animal health, especially during the period of the coronavirus disease 2019 (COVID-19) when increased use of masks and surgical gloves led to the generation of a large amount of medical waste [2,3,4,5].

Plastics are further degraded into fragments in the environment. Thompson et al. [6] first proposed microplastics (MPs), which were later defined as “plastic particles smaller than 5 mm” [7]. Meanwhile, plastic particles below 0.1 μm are called nanoplastics (NPs) [8]. MPs have been detected in water [9,10], soil [11,12], air [13,14,15], tap water [16,17,18], drinking water [19,20], and even human food [21,22,23,24,25]. The main types of MPs are polyethylene (PE), polypropylene (PP), and polystyrene (PS) [26]. MPs can move along the food chain to higher-level organisms, including humans. Oral intake is a major route for human exposure to MPs, and they have been detected in adult and infant feces [27,28,29] and human placenta [30,31], among which, PP constituted the highest proportion of MPs, and its relative mass abundance in adult feces was up to 61.0% [28]. In addition, infants who are fed formula from PP infant feeding bottles are exposed to PP-MPs ranging from 14,600–4,550,000 particles per capita per day, depending on the region [32]. Therefore, more attention should be paid to the influence of MPs (especially PP) on human health.

Most current reports on the toxicity of MPs focus on PS, while the biological safety assessment of PP is mainly concentrated on aquatic organisms [33,34,35] and plants [36,37]. Previous studies have reported that PP-MPs can reduce the thickness of the intestinal mucosa and intestinal muscle layer, cause oxidative stress and inflammation in intestinal tissue, and seriously interfere with lipid metabolism in zebrafish [38,39]. Ten micrograms per liter of 70 μm PP-MP significantly reduced the survival rate of zebrafish [40]. However, few studies have measured the effects of PP-MPs on mammalian and cell models, although PP-MPs contribute to pulmonary inflammation in vivo and affect the level of immune cytokines in vitro [41,42]. Meanwhile, PP-MPs with an average particle size of > 50 μm do not exhibit acute toxicity in rats [43,44,45]. However, there is no report on the biosafety of PP particles < 10 μm in the environment, thus warranting further study.

The intestinal tract may be the primary target organ after oral ingestion of PP-MPs. The intestinal barrier plays a key role in evaluating PP-MP intestinal toxicity and its toxic effects on distal tissues and organs. Orally ingested MPs can accumulate in organisms through the intestinal barrier and cause adverse effects such as flora imbalance and metabolic changes, which may lead to damage in multiple systems and organs [46,47,48]. Thus, MP exposure can cause intestinal barrier dysfunction or even damage, which may be a key factor for MP biotoxicity. However, the effect of orally ingested PP-MPs on the intestinal tract (especially on the intestinal barrier) remains unknown.

The mechanical and chemical barriers are two important aspects of the intestinal barrier. The mechanical barrier consists of intact intestinal epithelial cells and tight connections between cells. Goblet cells in the intestinal epithelium secrete mucus to form the intestinal mucus barrier, which is an important component of the chemical barrier [49,50]. The intestinal mucus layer covers almost the entire intestinal cavity surface; it lubricates, resists bacterial invasion, and protects the intestinal tract from mechanical damage and pathogenic bacteria [51,52]. Therefore, both the intestinal epithelial cell layer and mucus layer form the first line of defense against external factors and play an important role in maintaining the balance of the intestinal environment and blocking intestinal pathogens and toxins [53,54]. However, pathogenic factors, such as stress and inflammation, can destroy intestinal barrier function and cause intestinal mucosal barrier injury [55,56].

This study aimed to clarify the mechanism and effect of orally ingested PP-MPs on the intestinal tract. To this end, two kinds of PP-MP particles with different scales were selected, the relevant doses of environmental exposure were adopted, and a subacute oral ingestion model in mice was established to study the effects of PP-MPs on the intestinal mucosal barrier and the changes in related signaling pathways. Ultimately, we aimed to evaluate the intestinal toxicity caused by PP-MPs. This is the first study on the intestinal toxicity of ~10 μm PP-MPs in mice, which provides a toxicological reference for the biosafety assessment of environmental PP-MPs and their risk of exposure to humans.

## 2. Materials and Methods

### 2.1. Characterization of PP Particles and Suspension Preparation

**Figure 1:**
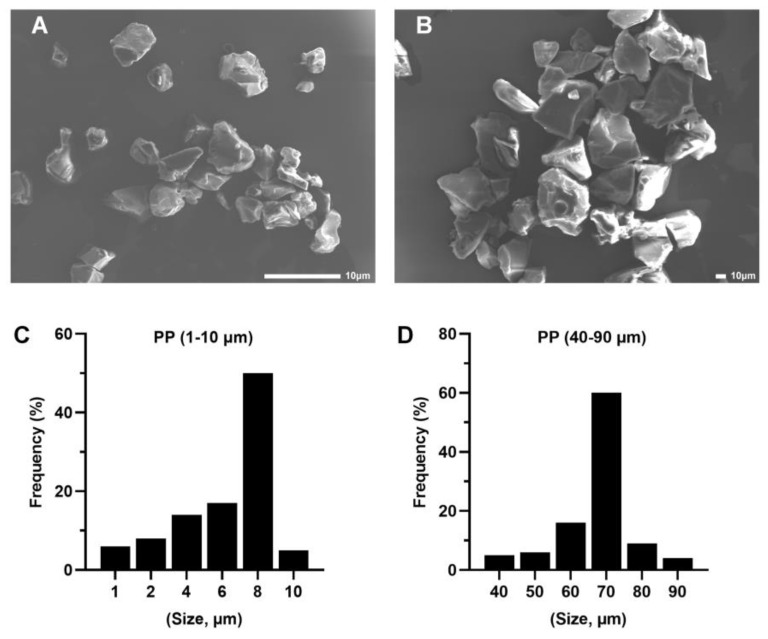
Characterization and scanning electron microscopy (SEM) images of polypropylene (PP) particles. (**A**) SEM images of 8 μm PP particles (scale bar = 10 μm) determined with a particle size analyzer (**C**). (**B**) SEM images of 70 μm PP particles (scale bar = 10 μm) determined with a particle size analyzer (**D**).

PP was purchased from Shanghai Macklin Biochemical Co., Ltd. (Shanghai, China), and PP-MP particles were prepared according to a previous method [41,57]. PP material was frozen in liquid nitrogen for 10 min and the powder was ground with a temperature-controlled three-dimensional vibration ball mill (TJSKW; Techin, Tianjin, China) at 4 °C for 25 min. The powder was collected and separated with an electromagnetic vibration sieve (TJ-TAS; Techin). The PP particles were separated by size using sieves with ~10 μm and 20–100 μm pore sizes. All of the PP particles were irregular blocks according to scanning electron microscopy (SEM) images (Tescan VEGA3; Tescan, Brno, Czech Republic) (Figure 1A,B). The particle size ranges of PP are 1–10 μm (50% are 8 μm) and 40–90 μm (60% are 70 μm) (NIH, Bethesda, MD, USA) [58] (*n* = 100) (Figure 1C,D). Therefore, to express the results of this study, we used 8 μm to represent the size of smaller PP particles and 70 μm to represent the size of larger PP particles.

A 10 mg/mL suspension of PP particles was prepared in pure water, followed by the addition of Tween 80 to 0.01% *v*/*v*. The mixture was ultrasonicated at 40K Hz for 1 hr using ultrasonic cleaner (DS-2510DTH; Shanghai Aucy Scientific Instrument Co., Ltd., Shanghai, China), then diluted to the desired concentration. The suspension was ultrasonicated for 30 min and fully mixed in the eddy current oscillator before administration to animals.

### 2.2. Animals and Experimental Design

Seventy-two male C57BL/6 mice of 22–26 g body weight were purchased from Beijing Vital River Laboratory Animal Technology Co., Ltd., China (animal production license number: SCXK (Beijing) 2016–0006). All animal experiments were approved by the Ethics Committee of Animal Care and Experimentation of the National Institute for Environmental Studies, China, IACUC approval code AMMS-04-2021-014. The mice were reared in a specific pathogen-free (SPF) animal room with an ambient temperature of 23 ± 2 °C, a photoperiod of 12 h light/12 h darkness, and a relative humidity of 40–60%. The diet contained bran, soybean meal, corn, flour, sorghum flour, fish meal, calcium hydrogen phosphate, and salt. All ingredients complied with the corresponding national food hygiene standards. Food and sterilized water were provided ad libitum. The exposure dose of PP-MPs (8 and 70 μm) was 1, 10, and 100 mg/kg/d; that is, the exposure concentration was 0.1, 1, and 10 mg/mL, respectively. This exposure dose is based on the previous literature: the intake of MPs for adult humans (calculated for 70 kg) is 0.1–5 g per week, or 0.2–10.2 mg/kg body weight (bw)/d [59]. Therefore, the PP dose selected for the test reflects the real range of human MP intake.

Mice were acclimatized for one week, then randomly divided into eight groups: blank control (pure water, BC), solvent control (pure water containing 0.01% *v*/*v* Tween-80, SC), 8 μm PP at 0.1 mg/mL (Ls), 8 μm PP at 1.0 mg/mL (Ms), 8 μm PP at 10 mg/mL (Hs), 70 μm PP at 0.1 mg/mL (Lb), 70 μm PP at 1.0 mg/mL (Mb), and 70 μm PP at 10 mg/mL (Hb). Each mouse group was housed in two cages, four or five in each cage. The mice were given 0.1 mL/10 g bw of PP-MPs suspension by oral gavage for 28 days. At the end of the experiment, mice were fasted for 12 h and anesthetized. Colon tissues were carefully isolated, colon segments of about 4 cm were taken, and then the intestinal contents were washed with aseptic PBS buffer. Some of the colon samples were fixed with different solutions for subsequent staining sections, and the remaining samples were frozen in liquid nitrogen for Western blotting and enzymatic analysis.

### 2.3. Histopathological Examination and Electron Microscopy Analysis

The colonic tissues of mice in each group were fixed in 4% *w*/*v* paraformaldehyde solution at 4 °C for 24 h, then dehydrated in gradient ethanol from 75% to 100% for 40 min, respectively, immersed in xylene to make it transparent, and embedded in paraffin wax. The embedded samples were cut into 3 μm thick sections with a microtome (RM2245; Leica, Nussloch, Germany) and stained with hematoxylin–eosin (H&E), and the histopathological changes were observed under an Olympus DP26 microscope (Tokyo, Japan).

The colonic tissues were soaked in 2.5% *w*/*v* glutaraldehyde at 4 °C for 24 h and fixed in 1% *w*/*v* osmic acid at 20 °C for 2 h. The samples were dehydrated with gradient ethanol from 30% to 100% for 20 min, respectively, and embedded in epoxy resin. Ultrathin sections (50 nm) were prepared with a microtome (UC7; Leica). The sections were double-stained with 2% *w*/*v* uranium acetate and lead citrate for 15 min at 20 °C and dried overnight. Ultrastructural changes were observed with a transmission electron microscope (Fei Tecnai G20 TWIN, FEI, Hillsboro, OR, USA).

### 2.4. Alcian Blue/Periodic Acid–Schiff (AB-PAS) Staining

Paraffin sections of the colonic tissues were prepared according to the method above. The sections were dewaxed in xylene, hydrated in a graded ethanol series (100%, 85%, and 75%), and stained with alcian blue/periodic acid–Schiff (AB-PAS). The dye solution was prepared according to the requirements of the kit (Nanjing JianCheng Bioengineering Institute, Nanjing, China). Then dye solution was added to the glass slide sample, incubated at room temperature (about 20 °C) for 8–15 min, washed with running water, dried naturally, and examined under a microscope. The mucus coverage ratio was calculated as the pixels in the mucus area to the total pixel area of the gut section. The pixels were determined using Image Pro Plus 6.0 software (Media Cybernetics, Rockville, MD, USA).

### 2.5. Immunohistochemical Analysis

Colon paraffin sections were taken for immunohistochemical analysis. The sections were dewaxed in xylene and hydrated in a graded ethanol series (100%, 85%, and 75%). Antigen retrieval was performed using sodium citrate antigen retrieval solution (pH 6.0), following the manufacturer’s instructions (Beijing Solarbio Science & Technology Co., Ltd., Beijing, China). The sections were incubated with 3% *v*/*v* hydrogen peroxide in the dark for 25 min to quench endogenous peroxidase activity. After blocking with normal goat serum (Solarbio) at room temperature (about 20 °C) for 30 min, sections were separately incubated with the following antibodies at 4 °C overnight: anti-solute carrier family 26 member 6 (SLC26A6, 1:400; Bioss, Beijing, China), anti-Na-K-2Cl cotransporter 1 (NKCC1, 1:200; CST, Boston, MA, USA), and anti-cystic fibrosis transmembrane conductance regulator (CFTR, 1:100; CST). Then, the sections were incubated with goat anti-rabbit IgG HRP secondary antibody (1:500; Sino Biological, Beijing, China) for 50 min at room temperature, stained with diaminobenzidine (DAB; Solarbio) for color development, and counterstained with hematoxylin (cell nucleus) for 8 min. Finally, the sections were dehydrated in ascending ethanol (75%, 85%, and 100%), hyalinized in xylene, and sealed with neutral gum. The positive areas of the sections were observed and photographed using an Olympus BX51 fluorescence microscope, and the fluorescence intensity was analyzed using with Image Pro Plus 6.0 software.

### 2.6. Detection of Oxidative Stress Markers

The colon tissues of each group of mice were prepared according to the kit requirements to detect the levels of colonic oxidative stress markers. The concentrations of reduced glutathione (GSH) and oxidized glutathione (GSSG) (GSH and GSSG Assay Kit, S0053, Beyotime Biotechnology Co., Ltd., Shanghai, China), malondialdehyde (MDA) (Lipid Peroxidation MDA Assay Kit, S0131S, Beyotime), and catalase (CAT) (Catalase assay kit (Visible light), A007-1-1, Jiancheng) were detected using colorimetric kits. Glutathione peroxidase (GSH-Px) expression was detected using a Total Glutathione Peroxidase Assay Kit with NADPH (S0058, Beyotime). Superoxide dismutase (SOD) content was determined using a Total Superoxide Dismutase Assay Kit with WST-8 (S0101S, Beyotime). Protein concentration was determined with a BCA Protein Assay Kit (Beyotime).

### 2.7. Enzyme-Linked Immunosorbent Assay (ELISA)

ELISA kits were used to determine the expression of inflammatory factors and intestinal-barrier-related proteins in colon tissues. Inflammatory factors included mouse tumor necrosis factor alpha (TNF-α; Mouse TNFα ELISA Kit, JL10484, Shanghai Jianglai Biotechnology Co., Ltd., Shanghai, China), interleukin 1 beta (IL-1β; Mouse IL-1β ELISA Kit, JL18442, Jianglai), interleukin 6 (IL-6; Mouse IL-6 ELISA Kit, JL20268, Jianglai), and interleukin 10 (IL-10; Mouse IL-10 ELISA Kit, JL20242, Jianglai). Intestinal-barrier-related proteins included mouse zonula occludens 1 (ZO-1; Mouse ZO-1 ELISA Kit, JL20409, Jianglai), occludin (Mouse Occludin ELISA Kit, JL20408, Jianglai), mucin-1 (MUC1; Mouse MUC1 ELISA Kit, JL26951, Jianglai), and claudin-1 (CLDN1; ELISA Kit for CLDN1, SEC388Mu, Wuhan Cloud Clone Technology Co., Ltd., Wuhan, China). The extracted colon homogenate was mixed with the reagent in the kits, followed by the addition of a chromogenic agent for color development, and finally treated with a stop solution according to the manufacturer’s protocols. The optical density (OD) of each sample was measured at 450 nm with a microplate reader (Molecular Devices M5E, Silicon Valley, Calif., USA). The levels of inflammatory factors and intestinal-barrier-related proteins were calculated using a standard curve. Protein concentration was determined with a BCA Protein Assay Kit (Beyotime).

### 2.8. Apoptosis Detection in the Colon

The paraffin sections of colonic tissue were generated as previously stated. The sections were dewaxed in xylene and hydrated with a graded ethanol series (100%, 85%, and 75%). A membrane-breaking working solution (PBS containing 0.5% Triton X-100) was added to cover the tissues. After being stained with TUNEL fluorescence staining solution (In Situ Cell Death Detection Kit-POD; Roche, Basel, Switzerland) at 37 °C for 2 h in the dark, the sections were washed with PBS (pH 7.4) three times, counterstained with DAPI (4′,6-diamidino-2-phenylindole) (cell nucleus) and sealed with anti-fluorescence quenching sealing tablets. Images of the sections were observed with an Olympus BX51 fluorescence microscope, and the number of apoptotic cells was counted. The TUNEL positive cell rate = number of apoptotic cells/total cells × 100% in each visual field.

### 2.9. Western Blot Analysis

Total protein extraction of colonic tissue was performed by treatment with RIPA buffer, followed by centrifugation at 12000 r/min for 5 min at 4 °C, and the concentration was measured with a BCA Protein Assay Kit (Beyotime). Proteins (50 μg) were electrophoresed in an 8–10% gradient sodium dodecyl sulfate polyacrylamide gel and transferred to polyvinylidene fluoride (PVDF) membranes (Millipore, Billerica, MA, USA). After blocking with 5% *w*/*v* nonfat milk dispersed in Tris-buffered saline containing 0.05% Tween-20 (TBST) for 1 h, the membranes were incubated with primary antibodies against IκBα (CST), p105 (CST), p50 (Abcam, Cambridge, UK), p65 (CST), p-p65 (CST), Toll-like receptor 4 (TLR4; Abcam), Bax (CST), Bcl-2 (CST), caspase-9 (CST), caspase-3 (CST), cleaved caspase-9 (CST) (all 1:1000), and cleaved caspase-3 (1:500; Bioss), as well as the internal control glyceraldehyde 3-phosphate dehydrogenase (GAPDH) antibody (1:2000; Tianjin UtiBody Biotechnology Co., Ltd., Tianjin, China) overnight at 4 °C. The membranes were washed with TBST (10 min wash × 3), followed by incubation with goat anti-rabbit IgG HRP secondary antibody (1:3000; Bioss) and goat anti-mouse IgG HRP (1:3000; Bioss) secondary antibody at room temperature (about 20 °C) for 1 hr. Membranes were washed three times with TBST, stained using ECL chemiluminescence reagent (Solarbio), and imaged in a fully automatic chemiluminescence image analysis system (Tanon 5200, Shanghai, China). The grayscale value of protein imprinting was analyzed using Gel-Pro analyzer software.

### 2.10. Statistical Analysis

Data are expressed as the mean ± standard deviation (SD). Statistical analyses were performed using SPSS 25.0 software. Differences between groups were determined according to one-way analysis of variance (ANOVA) followed by the least post-squares post hoc test for equal variances or Dunnett’s T3 post hoc test for unequal variances. Any *p*-value of <0.05 was considered to be statistically significant.

## 3. Results

### 3.1. Polypropylene Microplastic Exposure Changes Colonic Histopathology and Ultrastructure

**Figure 2:**
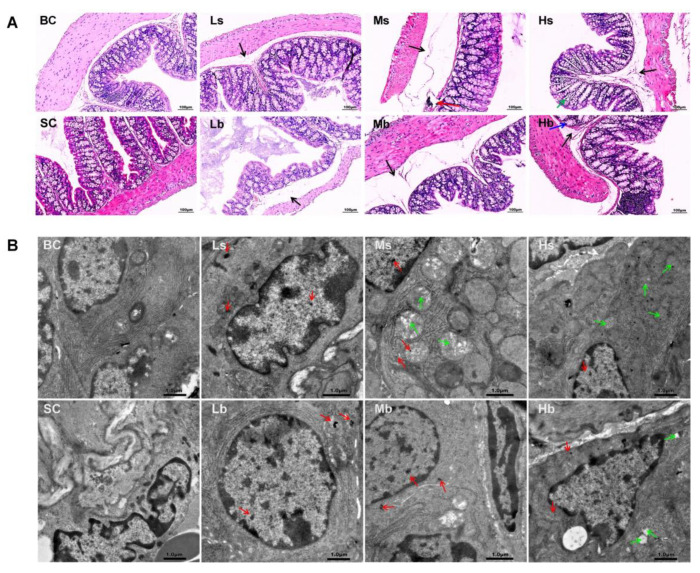
Effects of exposure to PP-MPs on colonic histopathology and ultrastructure in mice. (**A**) Images of H&E-stained colon sections of mice treated with PP-MPs. Black arrow: submucosal edema; red arrow: massive aggregation of lymphocytes; blue arrow: glandular hyperplasia of mucosal layer; green arrow: inflammatory cell infiltration. (**B**) Ultrastructural changes of colon after exposure to PP-MPs. Red arrows: PP particles; green arrow: slight abnormality of mitochondrial structure, with loose ridge arrangement, less matrix, partial swelling, and vacuolation. *n* = 3. BC: blank control (pure water); SC: solvent control (pure water containing 0.01% *v*/*v* Tween-80); PP-MPs: polypropylene microplastics. Ls: 8 μm PP at 0.1 mg/mL; Ms: 8 μm PP at 1.0 mg/mL; Hs: 8 μm PP at 10 mg/mL; Lb: 70 μm PP at 0.1 mg/mL; Mb: 70 μm PP at 1.0 mg/mL; Hb: 70 μm PP at 10 mg/mL. scale bar = 1 μm.

The whole-colon tissue structure of mice in the blank control group and solvent control group was basically normal, and there were no abnormalities in the mucosal layer and muscular layer. However, mild edema of the submucosa was found in the colon of mice in all PP-MP treatment groups. In addition, a large number of lymphocytes gathered in the colon of mice in the 1.0 mg/mL treatment group using 8 μm PP-MPs. A small amount of inflammatory cells infiltrated the colon of mice in the 10 mg/mL treatment group. Glandular hyperplasia of the mucosal layer appeared in the colon of mice in the 10 mg/mL treatment group using 70 μm PP-MPs (Figure 2A).

In addition, the ultrastructure of the colonic epithelial cells of mice was basically normal in the blank control group and the solvent control group; also, the nucleus was clearly visible, and the mitochondrial structure was normal. The ultrastructure of colonic epithelial cells of mice in all PP-MP treatment groups was also basically normal, and the nucleus was clearly visible. Meanwhile, PP granules were found in colonic epithelial cells of mice in all PP-MPs treatment groups. The ultrastructural changes of colonic epithelial cells of mice treated with 8 μm PP-MPs (1 and 10 mg/mL) and 70 μm PP-MPs (10 mg/mL) included mild abnormalities in the mitochondrial structure: loose ridge arrangement, reduced matrix, partial swelling, and vacuolation (Figure 2B).

### 3.2. Polypropylene Microplastic Exposure Induces Colonic Oxidative Stress and Inflammation

**Figure 3:**
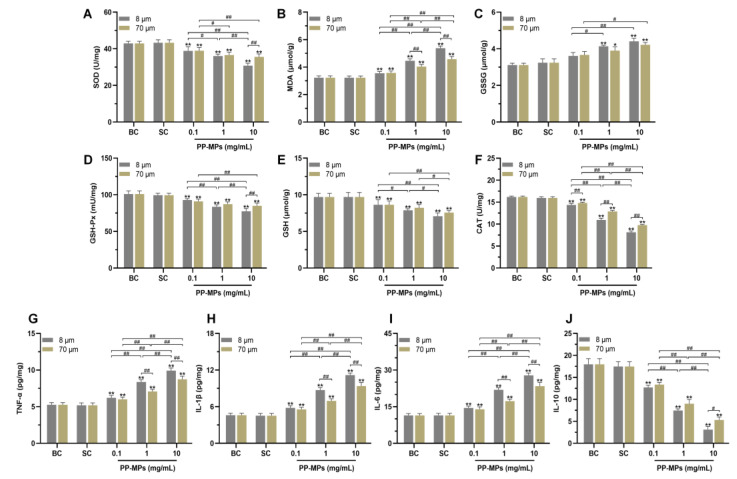
Oxidative stress and inflammation occurred in the colon of mice after PP-MP exposure. (**A**–**F**) Colonic levels of SOD (**A**), MDA (**B**), GSSG (**C**), GSH-Px (**D**), GSH (**E**), and CAT (**F**). (**G**–**J**) Colonic levels of TNF-α (**G**), IL-1β (**H**), IL-6 (**I**), and IL-10 (**J**). The presented values are the means ± SD (*n* = 5). *^#^ p* < 0.05, *^##^ p* < 0.01 between PP-MP treated groups, and * *p* < 0.05, ** *p* < 0.01 vs. SC as determined by one-way analysis of variance (ANOVA). BC: blank control (pure water); SC: solvent control (pure water containing 0.01% *v*/*v* Tween-80); PP-MPs: polypropylene microplastics.

There was no significant difference in the levels of MDA, GSSG, SOD, GSH, GSH-Px, and CAT in the colon of mice in the blank control group compared with that of the solvent control group (Figure 3A–F). The levels of SOD, GSH, GSH-Px, and CAT in all PP-MP treatment groups significantly decreased (*p* < 0.05) compared with that of the solvent control group, and the MDA level significantly increased (*p* < 0.05); each of these relationships showed a certain concentration dependence. Exposure to 8 μm and 70 μm PP-MPs at 1 mg/mL and 10 mg/mL significantly increased the level of GSSG compared with that in the solvent control group (*p* < 0.05). In addition, the expression of CAT in the 8 μm PP-MP treatment group was significantly lower than that in the 70 μm PP-MP treatment group (*p* < 0.05). The MDA level in the 8 μm PP-MP treatment group was significantly higher than that in the 70 μm PP-MP (1 and 10 mg/mL) treatment groups (*p* < 0.05). The levels of SOD and GSH-Px in the 8 μm PP-MP treatment group were significantly lower than those in the 70 μm PP-MP treatment group (*p* < 0.05) at the exposure concentration of 10 mg/mL. These results indicate that PP-MP exposure caused an imbalance in the redox system in the intestinal tissues of mice, resulting in oxidative damage.

There was no significant difference in the levels of TNF-α, IL-1β, IL-6, and IL-10 in the colon of mice in the blank control group compared with that of the solvent control group (Figure 3G–J). The levels of TNF-α, IL-1β, and IL-6 significantly increased in all PP-MP treatment groups, and IL-10 expression significantly decreased (*p* < 0.05) compared with that of the solvent control group. The effect was concentration-dependent. In addition, the levels of TNF-α, IL-1β, and IL-6 were significantly higher in the 8 μm PP-MP treatment group than those in the 70 μm PP-MP (1 and 10 mg/mL) treatment groups (*p* < 0.05). The level of IL-10 in the 8 μm PP-MP treatment group was significantly lower than that in the 70 μm PP-MP (10 mg/mL) treatment group (*p* < 0.05). These results suggest that inflammatory reactions occur in the colon of mice exposed to PP-MPs.

### 3.3. Polypropylene Microplastic Exposure Activates the TLR4/NF-κB Signaling Pathway

**Figure 4:**
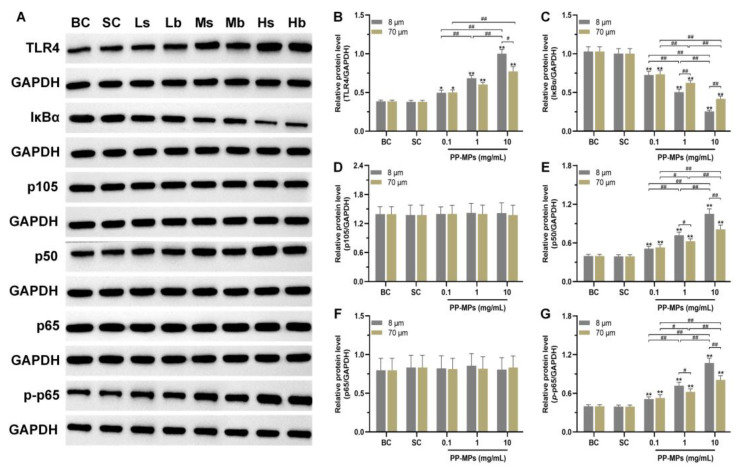
Effects of PP-MP exposure on protein expression of the TLR4/NF-κB signaling pathway. (**A**) Western blot of TLR4, p105, p50, p65, *p*-p65, and IκBα protein expression. GAPDH was used as an internal reference. (**B**–**G**) Quantitative expression of these proteins. The values are the means ± SD (*n* = 4). *^#^ p* < 0.05, *^##^ p* < 0.01 between PP-MP treated groups, and * *p* < 0.05, ** *p* < 0.01 vs. SC as determined by one-way analysis of variance (ANOVA). BC: blank control (pure water); SC: solvent control (pure water containing 0.01% *v*/*v* Tween-80); PP-MPs: polypropylene microplastics.

The levels of TLR4, p105, p50, p65, *p*-p65, and IκBα were not significantly different in the colon of the blank control mice from those in the solvent control group (Figure 4). The levels of TLR4, p50, and *p*-p65 significantly increased in all PP-MP treatment groups, and IκBα expression significantly decreased (*p* < 0.05) compared with that of the solvent control group. The differences were all concentration-dependent. In addition, the level of IκBα was significantly lower in the 8 μm PP-MP treatment group than that in the 70 μm PP-MP (1 and 10 mg/mL) treatment groups (*p* < 0.05). Also, the levels of p50 and *p*-p65 in the 8 μm PP-MP treatment group were significantly higher than that in the 70 μm PP-MP treatment group (*p* < 0.05). The level of TLR4 in the 8 μm PP-MP treatment group was significantly higher than that in the 70 μm PP-MP (10 mg/mL) treatment group at (*p* < 0.05).

### 3.4. Polypropylene Microplastic Exposure Destroys the Intestinal Mucosal Barrier in Mice

**Figure 5:**
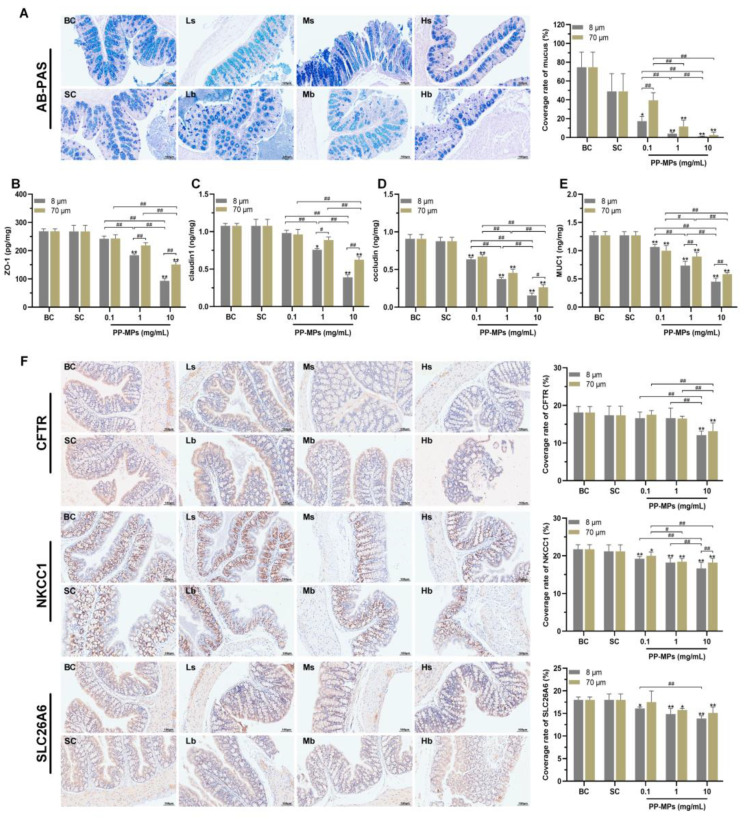
Polypropylene microplastics affect the intestinal barrier in mice. (**A**) AB-PAS staining and the ratio of the mucus coverage area to the entire colon area. Glycogen and neutral mucopolysaccharides in tissues are red, neutral mucus and acidic mucus are purple, and the nucleus is blue. (**B**–**E**) The expression of ZO-1 (**B**), claudin-1 (**C**), occludin (**D**), and MUC1 (**E**) in the colon. (**F**) Immunohistochemistry of the colonic ion channel transport protein CFTR, NKCC1, and SLC26A6. The nucleus stained with hematoxylin is blue and the positive expression is brown. The values are the means ± SD (normalized amounts of AB-PAS and immunohistochemistry, *n* = 3; intestinal-barrier-related protein level, *n* = 5). *^#^ p* < 0.05, *^##^ p* < 0.01 between PP-MP treated groups, and * *p* < 0.05, ** *p* < 0.01 vs. SC as determined by one-way analysis of variance (ANOVA). BC: blank control (pure water); SC: solvent control (pure water containing 0.01% *v*/*v* Tween-80); PP-MPs: polypropylene microplastics.

AB-PAS staining showed that there was no significant difference in the colonic mucus coverage rate between the blank control group and the solvent control group (Figure 5A). The colonic mucus coverage rate of mice treated with 8 μm and 70 μm PP-MPs (1 and 10 mg/mL) significantly decreased compared with that of the solvent control group (*p* < 0.05).

The levels of ZO-1, claudin-1, occludin, and mucin (MUC1) were not significantly different in the colon of mice in the blank control group from those in the solvent control group (Figure 5B–E). The levels of occludin and MUC1 in all PP-MP treatment groups significantly decreased (*p* < 0.05) in a concentration-dependent manner compared with that of the solvent control group. The levels of ZO-1 and claudin-1 significantly decreased in the 8 μm PP-MP (1 and 10 mg/mL) treatment groups and 70 μm PP-MP (10 mg/mL) treatment group compared with that of the solvent control group (*p* < 0.05). In addition, the expression of ZO-1, claudin-1, and MUC1 were significantly lower in the 8 μm PP-MP treatment group than those in the 70 μm PP-MP treatment group (*p* < 0.05) at 1 mg/mL and 10 mg/mL exposure concentrations. Occludin expression was significantly lower in the 8 μm PP-MP treatment group than that in the 70 μm PP-MP treatment group (*p* < 0.05) at the exposure concentration of 10 mg/mL.

Immunohistochemical analysis of the colon showed that there was no significant difference in the levels of CFTR, SLC26A6, and NKCC1 between the blank control group and the solvent control group (Figure 5F). The level of NKCC1 significantly decreased in all PP-MP treatment groups (*p* < 0.05) compared with that of the solvent control group. The level of SLC26A6 significantly decreased in the 8 μm PP-MP (0.1, 1, and 10 mg/mL) treatment groups and 70 μm PP-MP (1 and 10 mg/mL) treatment groups compared with that of the solvent control group (*p* < 0.05). The level of CFTR was significantly lower in the 8 μm and 70 μm PP-MP treatment groups than that in the solvent control group (*p* < 0.05) at the exposure concentration of 10 mg/mL.

### 3.5. Polypropylene Microplastic Exposure Promotes Apoptosis of Colonic Cells

**Figure 6:**
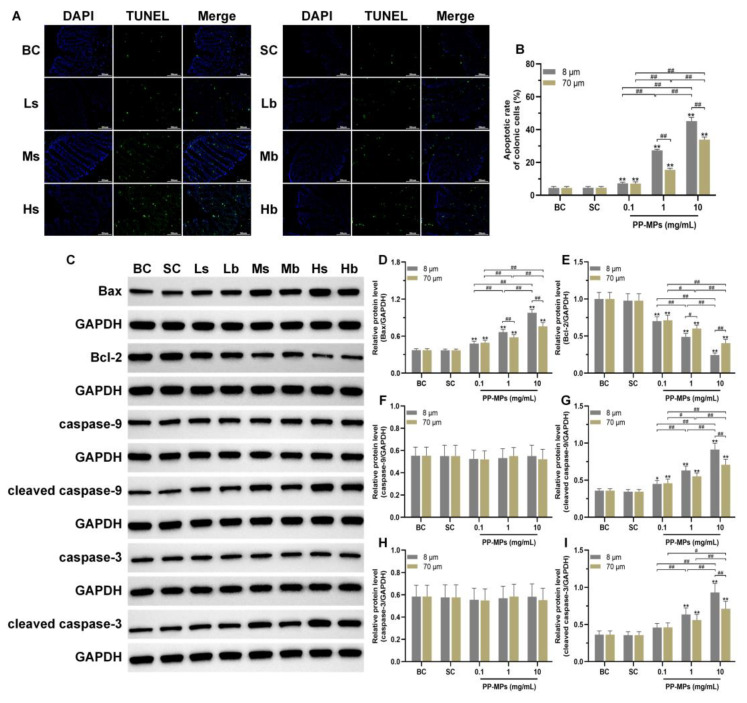
Effects of exposure to PP-MPs on apoptosis and the apoptosis pathway in colon tissue. (**A**) Images of colon sections stained with TUNEL to assess cell apoptosis after PP-MP exposure (200×). Apoptotic cells are green, and the nucleus was stained with DAPI in blue. (**B**) Quantification of the apoptotic rate of colonic cells. (**C**) Western blot of Bax, Bcl-2, caspase-9, caspase-3, cleaved caspase-9, and cleaved caspase-3. (**D**–**I**) Quantitative expression of these proteins. The presented values are the means ± SD (TUNEL staining: *n* = 3; Western blot analysis: *n* = 4). *^#^ p* < 0.05, *^##^ p* < 0.01 between the PP-MP treated groups, and * *p* < 0.05, ** *p* < 0.01 vs. SC as determined by one-way analysis of variance (ANOVA). BC: blank control (pure water); SC: solvent control (pure water containing 0.01% *v*/*v* Tween-80); PP-MPs: polypropylene microplastics.

The TUNEL experiment showed that there was no significant difference in the apoptotic rate of colonic cells between the blank control group and the solvent control group (Figure 6A,B). The apoptotic rate of colonic cells significantly increased (*p* < 0.05) in all PP-MP treatment groups in a concentration-dependent manner compared with that of the solvent control group. In addition, the apoptotic rate of colonic cells was significantly higher in the 8 μm PP-MP treatment group than that in the 70 μm PP-MP (1 and 10 mg/mL) treatment groups (*p* < 0.05).

Furthermore, there was no significant difference in the levels of Bax, Bcl-2, caspase-9, cleaved caspase-9, caspase-3, and cleaved caspase-3 (all proteins related to apoptosis) in the colon of mice in the blank control group compared with the solvent control group (Figure 6C–I). Bcl-2 levels significantly decreased in all PP-MP treatment groups, while the levels of Bax and cleaved caspase-9 significantly increased (*p* < 0.05) compared with the solvent control group; each change was concentration-dependent. The levels of cleaved caspase-3 were significantly higher (*p* < 0.05) in the 8 μm and 70 μm PP-MP treatment groups (1 and 10 mg/mL) than those in the solvent control group. At the same time, the Bax level was significantly higher after 8 μm PP-MP treatment than after 70 μm PP-MP treatment, while the Bcl-2 level was significantly lower after 8 μm PP-MP treatment compared with that of the 70 μm PP-MP treatment group (*p* < 0.05) at exposure concentrations of 1 mg/mL and 10 mg/mL. The levels of cleaved caspase-9 and cleaved caspase-3 were significantly higher in the 8 μm PP-MP treatment group than those in the 70 μm PP-MP treatment group (*p* < 0.05) at the exposure concentration of 10 mg/mL.

## 4. Discussion

There is little research on the biosafety of PP-MPs in mammals. This study evaluated the intestinal toxicity and possible mechanism of subacute oral ingestion of PP-MPs (8 μm and 70 μm) in mice. These sizes were selected because MPs with a particle size of 1–50 μm have been found to be dominant in tap water samples collected from consumer homes [18]. Moreover, MPs (<10 μm) can be absorbed by intestinal epithelial cells and accumulate in the intestine, after which they pass through the intestinal barrier and enter the systemic circulation to reach other organs [60,61]. MP fragments with sizes ranging between 5–10 μm were found in the placenta after vaginal delivery; the most common type of detected MP was PP [30], while those with sizes above 50 μm were found in the placenta and meconium post-cesarean section delivery [31].

According to our pathological and electron microscopy results, we speculate that PP-MPs may enter the intestinal epithelial cells through the damaged intestinal mucosal barrier, after which they occur in the colon. In addition, we found that the 10 mg/mL treatment group exhibited the most severe colon injury at 8 μm PP-MP or 70 μm PP-MP. Inflammatory and mucosal injury in colon tissue and mitochondrial injury in the epithelial cell ultrastructure are typically related to oxidative stress [62,63]. Therefore, we measured indicators of oxidative stress and inflammation in the colon. Notably, damage caused by 8 μm PP-MP to the colon tissue of mice was more severe than that by 70 μm PP-MP at the exposure concentration of 1 mg/mL, and we propose that the particle size of PP affects its toxicity.

Our study also found that the levels of the antioxidant GSH and the antioxidant enzymes SOD, CAT, and GSH-Px significantly decreased in the colon of mice after PP-MP exposure, while MDA levels significantly increased. Among them, GSH plays an important role in scavenging reactive oxygen species, and GSH-Px directly or indirectly plays the role of the antioxidant [64]. MDA is the product of lipid peroxidation which can be used as an index to measure oxidative damage [65]. These results show that PP-MP exposure reduces the level of antioxidant defense in the colon tissue of mice, resulting in increased oxidative stress reactions. Simultaneously, the levels of the pro-inflammatory factors TNF-α, IL-1β, and IL-6 significantly increased in colon tissue after oral ingestion of PP-MPs, while the level of anti-inflammatory factor IL-10 significantly decreased, indicating the occurrence of inflammatory injury in the colon of mice. Our results are consistent with those of previous studies showing that MPs induce oxidative stress and inflammation [66,67,68]. In addition, the colonic oxidative stress and inflammatory damage was more severe in mice treated with 8 μm PP-MPs than in those treated with 70 μm PP-MPs at an exposure concentration of 10 mg/mL. Therefore, we speculate that oxidative stress and inflammatory injury play a key role in intestinal injury caused by oral ingestion of PP-MPs.

Pathological conditions such as inflammation or disorder of the redox system can increase tyrosine phosphorylation of occludin protein and change the interaction between occludin and ZO-1 protein, thus breaking the tight junction [69]. Tight junctions are mainly composed of occludin and claudin proteins, which are anchored to the actin cytoskeleton by the scaffold protein Zos [70]. ZO-1 is closely related to other tight junction proteins and functions as an adaptor that connects transmembrane proteins to the perijunctional actomyosin ring [71,72]. Occludin is a tight junction transmembrane protein that plays a key role in maintaining the tight junction barrier [73]. Claudin is the main determinant of tight junction paracellular permeability, and changes in claudin protein may lead to intestinal instability, inflammation, and the progression of necrotizing enterocolitis [74,75]. Destruction of the tight junction increases intestinal permeability, and harmful substances such as bacteria and endotoxins enter the systemic milieu, adversely affecting the health of the organism [76,77]. Rao et al. [78] believe that oxidative stress induces tight-junction destruction and increases intestinal permeability by mediating tyrosine phosphorylation and the redistribution of occluding–ZO-1 and E-cadherin–beta-catenin complexes from the intracellular junctions. In addition, TNF and interleukin (IL) can significantly disrupt the expression and distribution of tight junction proteins, thus damaging intestinal barrier function [79,80]. TNF-α increases intestinal permeability by affecting occludin internalization and interferes with ZO-1 subcellular localization and protein expression by stimulating NF-κB signal transduction, resulting in damage to intestinal barrier function [81,82]. Incubation of the intestinal epithelial cell monolayer (Caco-2 and T84) with IFN-γ and TNF-α promotes the recombination of tight junction proteins including ZO-1, occludin, and claudin-1, and reduces epithelial barrier function [83]. IL-1, IL-6, and IL-10 have a great influence on epithelial and endothelial paracellular permeability [82]. IL-1 intervention in vitro increases the permeability of intestinal epithelial tight junctions and downregulates occludin expression by activating NF-κB. In vitro IL-6 intervention increases the permeability across endothelial cells and leads to ZO-1 mislocalization. Meanwhile, IL-10 blocks epithelial permeability induced by IFN-γ to maintain the epithelial barrier and chloride secretion function; IL-10-deficient mice exhibit increased intestinal permeability [84,85,86,87]. We found that occludin expression significantly decreased in all PP-MP treatment groups, and the expression of ZO-1 and claudin-1 significantly decreased in the 8 μm and 70 μm PP-MP treatment groups at 10 mg/mL exposure concentration. This indicates that tight junctions were broken. In addition, the colonic tight junction injury in mice treated with 8 μm PP-MPs was more severe than in mice treated with 70 μm PP-MPs at 10 mg/mL exposure concentration. Therefore, we conclude that PP-MPs destroy the tight junctions of mice intestinal epithelium by mediating colonic oxidative stress and inflammation and induce intestinal mucosal barrier injury.

The mucus layer on the surface of the intestinal cavity (and secreted mucins) protects the intestinal mucosa and resists bacterial invasion [88]. MUC1 is a cell-surface mucin of intestinal epithelial cells that protects epithelial cells and participates in signal transduction [89,90]. Our results show that colonic mucus secretion and MUC1 expression significantly decreased in the 8 μm and 70 μm PP-MP treatment groups at exposure concentrations of 1 mg/mL and 10 mg/mL. Similarly, oral ingestion of PS nano/microplastics reduces mucus secretion and the level of *Muc1* gene transcription in the colon of mice [55,91], and exposure to PS-MPs through drinking water also resulted in such effects in the colon of mice [47,92]. Oxidative damage can induce mucin degradation, and higher ROS levels can reduce mucus barrier thickness [93]. TNF-α causes the loss of mucin-producing goblet cells by inducing the death of intestinal epithelial cells, while also affecting mucus layer composition by regulating the expression of other components in the mucus [94]. IL-10 promotes the production of intestinal mucus by inhibiting protein misfolding and endoplasmic reticulum stress in goblet cells, thus maintaining the mucus barrier [95]. Our results indicate that oral ingestion of PP-MPs causes oxidative damage and changes the levels of inflammatory cytokines in the intestinal tract of mice which further induces destruction of the intestinal mucus barrier.

Tight junctions maintain the intestinal epithelial barrier and regulate the osmotic gradients required for ion transport [96]. In particular, epithelial anion secretion can ensure the correct ion environment needed for the formation of a normal mucus layer [97,98,99]. We speculate that the ion transport process may be affected after intestinal barrier injury in mice. Our results show that the expression of chloride channel proteins in the colon significantly changed after PP-MP exposure, and NKCC1 expression significantly decreased in all PP-MP treatment groups. The levels of CFTR and SLC26A6 significantly decreased in the 8 μm and 70 μm PP-MP treatment groups at 10 mg/mL exposure concentration. CFTR is a chloride channel located in the apical membrane of cells; it plays a key role in regulating intestinal epithelial secretion, and its dysfunction leads to abnormal bacterial colonization [96,100,101]. NKCC1 provides chlorine for apical secretion through CFTR by mediating chloride uptake at the basolateral pole of intestinal epithelial cells [96]. SLC26 is a conservative anion transporter family, and SLC26A6 has the most extensive exchange function in the family; it mediates the transport of Cl^−^/HCO_3_^−^ and other anions, and plays an important role in ion homeostasis and acid-base balance [102]. Therefore, our results show that PP-MP exposure affects ion transport in the intestinal tract of mice. This is suggestive of damage to the intestinal barrier and mucus layer, which may cause pathogens and other harmful substances to enter blood circulation through the intestinal wall [103,104,105]. PP particles likely enter intestinal epithelial cells through the damaged intestinal barrier and deposit in the cells. This is consistent with the results observed using electron microscopy in this study.

TLR4 is the key receptor of intestinal innate immunity. It is expressed on a variety of cell surfaces of the intestinal mucosa and plays a key role in inducing inflammatory responses and producing inflammatory mediators [106]. NF-κB participates in the expression and regulation of various genes, plays a role in inflammation and immune response, and is a common transcription factor in the process of inflammation mediated by TLR4. NF-κB is composed of five members: p50 (p105), p52 (p100), p65, RelB, and c-Rel. The active form of p50 is formed by p105 proteolysis, and the signal transduction pathway triggered by inflammatory stimulation usually leads to the release and nuclear translocation of the NF-κB p50/p65 dimer [107,108,109]. In this study, there was no significant change in p105 and p65 levels in the colon of all PP-MP treated mice. However, the levels of TLR4, p50, and *p*-p65 significantly increased, and the level of IκBα significantly decreased. This indicates that PP-MPs induce a colonic inflammatory response through the TLR4/NF-κB signaling pathway. In addition, the level of IκBα was significantly lower in the 8 μm PP-MP treatment group than in the 70 μm PP-MP treatment group at an exposure concentration of 10 mg/mL, while the levels of TLR4, p50, and *p*-p65 were significantly higher than those in the 70 μm PP-MP treatment group. This indicates that 8 μm PP-MPs had a stronger effect on inducing intestinal inflammation in mice. TLR4 recruited MyD88 and further recruited the downstream kinase IRAK4 and the ubiquitin ligase TRAF6. This led to the activation of TAK1 and IκB phosphorylation, thus releasing the NF-κB p50/p65 dimer. The released dimer is translocated into the nucleus through modification processes such as phosphorylation. This induces NF-κB-mediated transcription of inflammatory cytokines, especially the increase in pro-inflammatory cytokines TNF-α, IL-1β, and IL-6 [109,110,111,112,113], which is consistent with the changes in inflammatory factors in this study.

Disorder of the redox system and inflammation can promote apoptosis [114,115]. In this study, colonic epithelial cells of mice showed mitochondrial damage after PP-MP exposure, and TUNEL results suggested apoptosis of colonic epithelial cells. We further detected proteins related to apoptosis in the mitochondrial pathway. PP-MP exposure significantly upregulated the levels of the apoptosis markers Bax, cleaved caspase-9, and cleaved caspase-3, and significantly downregulated the level of anti-apoptosis factor Bcl-2. Intracellular stress induces intrinsic apoptosis which depends on the process of mitochondrial outer membrane permeabilization. The process is mediated by the Bcl-2 family (including the pro-apoptotic proteins Bax and Bak and anti-apoptotic proteins Bcl-2, Bcl-xL, and MCL-1), which can release the contents of the mitochondrial intermembrane space (such as cytochrome C) into the cytoplasm. Cytochrome C interacts with apoptosis protease-activating factor 1 (Apaf-1), thus activating caspase-9 and triggering the cascade activation of caspase-3 and caspase-7 [116,117,118,119,120]. Caspase-3 is a terminal caspase protein that plays a vital role in cell apoptosis [121]. NF-κB can also activate apoptosis by upregulating the pro-apoptotic factor, Bax [122,123]. Further, we found that the levels of Bax, cleaved caspase-9, and cleaved caspase-3 were significantly higher in the 8 μm PP-MP treatment group than in the 70 μm PP-MP treatment group at an exposure concentration of 10 mg/mL. Moreover, the Bcl-2 level was significantly lower in the 8 μm PP-MP treatment group than in the 70 μm PP-MP treatment group. This indicates that 8 μm PP-MPs had a stronger effect on inducing colonic cell apoptosis in the mitochondrial pathway. These results indicate that PP-MPs induce endogenous apoptosis in the colon via the Bax/Bcl-2/Caspase-3 signaling pathway. Abnormal apoptosis in colonic cells (especially epithelial cells) causes damage to the intestinal mucosal barrier by reducing barrier components, and may reduce mucus production by reducing the number of goblet cells, which further destroys the intestinal barrier.

The particle size of MPs may affect their biotoxicity. Our results indicated that the toxicity of 8 μm PP-MP to the colon of mice was stronger than that of 70 μm PP-MP, especially at an exposure concentration of 10 mg/mL. Similarly, PP particles with diameters < 20 μm (dispersed in DMSO) are more toxic to macrophages than PP particles with diameters of 25–200 μm [41]. Compared with larger particles, smaller particles have a higher bioreactivity due to their higher specific surface area [60], which may be the main reason for the stronger toxic effects of smaller PP particles. This means that 8 μm PP-MP is more likely to come into contact with the intestine and exerts stronger irritation effects on the colon, which may lead to more severe damage to the intestinal mucosal barrier. Moreover, smaller MP particles may elicit stronger cytotoxicity compared to the larger particles due to the different absorption modes of intestinal epithelial cells [124]. A comparison of the toxic mechanism of MPs with different particle size scales requires further study. PS particles (< 10 μm) can activate p38, MAPK, Wnt/β-catenin, and other signaling pathways to exhibit toxicity [125,126,127,128]. However, there are few reports on the mechanism of toxicity of larger MP particles. In addition, with respect to the shape of MPs, the PP particles used in this study were irregular. Irregular and regularly shaped (spherical) MPs, such as PP and PS, can exert a variety of adverse effects by disrupting the balance of the redox system of organisms [34,39,125,126]. Therefore, the contribution of the shape to the biotoxicity of PP particles requires further research.

## 5. Conclusions

This study preliminarily demonstrated that oral ingestion of PP-MPs induces an imbalance in the redox system and activates the TLR4/NF-κB inflammatory signal pathway in the mouse intestine. This induces oxidative stress, inflammation, and apoptosis of intestinal epithelial cells through the mitochondrial pathway, which leads to intestinal barrier dysfunction and intestinal mucosal barrier damage, resulting in intestinal toxicity. Smaller PP-MPs exhibit higher intestinal toxicity compared with larger PP-MPs at the same exposure dose. This study provides data and reference for biological risk assessment of exposure to environmental PP-MPs via oral ingestion in mice. The findings can further aid in the formulation of pollution prevention and control policies for plastics.

## Data Availability

The data presented in this study are available on request from the corresponding author. The data are not publicly available due to privacy.
