# Correction: Jia et al. Exposure to Polypropylene Microplastics via Oral Ingestion Induces Colonic Apoptosis and Intestinal Barrier Damage through Oxidative Stress and Inflammation in Mice. *Toxics* 2023, *11*, 127

**DOI:** 10.3390/toxics11090733

**Published:** 2023-08-25

**Authors:** Rui Jia, Jie Han, Xiaohua Liu, Kang Li, Wenqing Lai, Liping Bian, Jun Yan, Zhuge Xi

**Affiliations:** 1 College of Marine Ecology and Environment, Shanghai Ocean University, Shanghai 201306, China; jrui1115@hotmail.com; 2 Tianjin Institute of Environmental and Operational Medicine, Tianjin 300050, China; hanjie0304@foxmail.com (J.H.);

## Figure Legend

In the original publication [1], there was a mistake in the legend for Figure 5. The description of the AB-PAS staining results in Figure 5A was not accurate. The correct legend appears below. 

**Figure 5.** Polypropylene microplastics affect the intestinal barrier in mice. (**A**) AB-PAS staining and the ratio of the mucus coverage area to the entire colon area. Neutral mucin is purplish red, and acidic mucin is blue; the tissues and cells containing both neutral and acidic mucins show different degrees of purple. (**B**–**E**) The expression of ZO-1 (**B**), claudin-1 (**C**), occludin (**D**), and MUC1 (**E**) in the colon. (**F**) Immunohistochemistry of the colonic ion channel transport proteins CFTR, NKCC1, and SLC26A6. The nucleus stained with hematoxylin is blue and the positive expression is brown. The values are the means ± SDs (normalized amounts of AB-PAS and immunohistochemistry, *n* = 3; intestinal-barrier-related protein level, *n* = 5). ^#^ *p* < 0.05, ^##^ *p* < 0.01 between PP-MP treated groups, and * *p* < 0.05, ** *p* < 0.01 vs. SC was determined via one-way analysis of variance (ANOVA). BC: blank control (pure water); SC: solvent control (pure water containing 0.01% *v*/*v* Tween-80); PP-MPs: polypropylene microplastics.

## Error in Figure

In the original publication [1], there was a mistake in Figure 6I as published. In Figure 6I, the significant marker of the cleaved caspase-3 protein expression level of 70 μm PP-MPs between treatment groups (0.1, 1 and 10 mg/mL) was not accurate. The corrected Figure 6 appears below.

## Text Correction

There was an error in the original publication [1]. The name of the animal ethics committee was misstated in the article. There are two places that must be revised.

In Section 2.2, Animals and Experimental Design, in the first paragraph, lines 3–5, we make the following correction:

“All animal experiments were approved by the Experimental Animal Welfare Ethics Committee of the Tianjin Institute of Environmental and Operational Medicine, IACUC approval code AMMS-04-2021-014.”

In the Institutional Review Board Statement, in the first paragraph, we make the following correction:

“The animal study protocol was approved by the Experimental Animal Welfare Ethics Committee of the Tianjin Institute of Environmental and Operational Medicine, IACUC approval code AMMS-04-2021-014. Approval date: 11 April 2021.”

The authors state that the scientific conclusions are unaffected. This correction was approved by the Academic Editor. The original publication has also been updated.

## Figures and Tables

**Figure 6:**
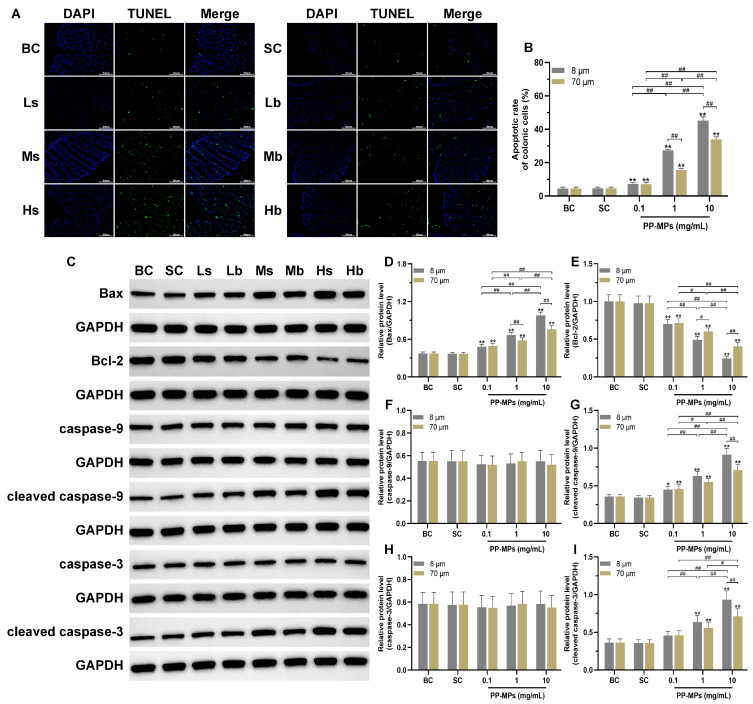
Effects of exposure to PP-MPs on apoptosis and the apoptosis pathway in colon tissue. (**A**) Images of colon sections stained with TUNEL to assess cell apoptosis after PP-MP exposure (200×, scale bar = 50 μm). Apoptotic cells are green, and the nucleus was stained with DAPI in blue. (**B**) Quantification of the apoptotic rate of colonic cells. (**C**) Western blot of Bax, Bcl-2, caspase-9, caspase-3, cleaved caspase-9, and cleaved caspase-3. (**D**–**I**) Quantitative expression of these proteins. The presented values are the means ± SDs (TUNEL staining: *n* = 3; Western blot analysis: *n* = 4). ^#^ *p* < 0.05, ^##^ *p* < 0.01 between the PP-MP treated groups, and * p < 0.05, ** p < 0.01 vs. SC was determined via one-way analysis of variance (ANOVA). BC: blank control (pure water); SC: solvent control (pure water containing 0.01% *v*/*v* Tween-80); PP-MPs: polypropylene microplastics.
